# Experiences using an online therapist-guided psychotherapy platform (OPTT) in correctional workers with depression, anxiety, and PTSD

**DOI:** 10.3389/fpsyt.2024.1365746

**Published:** 2024-04-23

**Authors:** Elnaz Moghimi, Gilmar Gutierrez, Callum Stephenson, Tessa Gizzarelli, Jasleen Jagayat, Christina Holmes, Charmy Patel, Mohsen Omrani, Alexander Ian Frederic Simpson, Nazanin Alavi

**Affiliations:** ^1^Department of Psychiatry, Faculty of Health Sciences, Queen’s University, Kingston, ON, Canada; ^2^Waypoint Research Institute, Waypoint Centre for Mental Health Care, Penetanguishene, ON, Canada; ^3^Centre for Neuroscience Studies, Faculty of Health Sciences, Queen’s University, Kingston, ON, Canada; ^4^OPTT Inc., Toronto, ON, Canada; ^5^Centre for Addiction and Mental Health, Toronto, ON, Canada

**Keywords:** correctional workers, public safety personnel, digital mental health interventions, mental health disorders, online psychotherapy, internet, user experience, participant experiences

## Abstract

**Introduction:**

Correctional workers (CWs) are frequently exposed to potentially traumatic events in the workplace, leading to an increased prevalence of mental health concerns. Online psychotherapy can address many of the barriers CWs face when seeking adequate mental health care. Despite their benefits, CWs’ experience using digital mental health interventions is relatively unknown. This information could be valuable in developing enhanced care delivery to improve recruitment, retention, satisfaction, and treatment outcomes.

**Methods:**

This study investigated the experiences of a sample of CWs enrolled in a clinical trial evaluating the efficacy of the Online Psychotherapy Tool (OPTT) in this population. Participants were surveyed and interviewed to capture their opinions and feedback on the program. Survey analysis was conducted through Qualtrics statistical analysis software. The interview transcripts and open-ended survey questions were analyzed using thematic analysis methods in NVivo.

**Results:**

Participants (n=14) were cis-gender, predominantly white, with an average age of 38 years. While most respondents preferred in-person therapy, they also reported the benefits of the online psychotherapy program. Specifically, they expressed positive perceptions of the platform, the quality and interaction of their care provider, and the homework assignments and skills learned. Lack of motivation to complete weekly homework assignments was a frequently cited challenge. Unhelpful aspects of the therapy noted issues with the online format and frustration with certain program elements.

**Discussion:**

Participants expressed a positive outlook on the program, the platform, and treatment outcomes. A preference for in-person therapy was still indicated, demonstrating the need to focus on engagement in digital mental health interventions. In addition, the findings of this study shed light on the factors that can influence help-seeking in this population, including stigma in the work environment, demanding work schedules, workplace perceptions, and previous experiences accessing mental health services.

## 1 Introduction

Correctional workers (CWs) are public safety personnel (PSP) tasked with overseeing the safety, security, and service delivery for staff and offenders within prisons, jails, courthouses, and correctional centers (1). CWs are frequently exposed to potentially psychologically traumatic experiences in their work environments that can compromise their mental health (2). Previous research has reported that half of correctional officers meet the diagnostic criteria for one or more mental health disorders (3). CWs often face barriers to accessing and obtaining mental health support (4). For instance, in-person cognitive behavioral therapy (CBT) may have limitations pertaining to geographic location, lack of time due to demanding work schedules, privacy concerns and stigma (5). Comparatively, online CBT (e-CBT) has the potential to offer a more accessible, convenient, and confidential method of treatment delivery. However, its use in CWs is in the nascent phase.

As of 2021, a clinical trial is being conducted to assess the efficacy of the Online Psychotherapy Tool (OPTT) in managing psychiatric symptoms experienced by CWs (6). OPTT is a secure and interactive cloud-based online platform that offers multiple diagnosis-specific care provider-guided asynchronous treatment programs, including e-CBT (7). Capturing participant experiences with the therapy platform and content is important in this ongoing clinical trial for continuous program evaluation and improvement. Although e-CBT adheres to the same principles as traditional in-person CBT, the novelty of this therapy format underscores the importance of exploring factors that enhance its effectiveness. Factors related to participant representation within the modules, user-friendliness, care provider interactions, and trauma-informed content have been previously reported by OPTT users to impact engagement (8). Before adapting the e-CBT programs for CWs, qualitative data from our research team also indicated that CWs viewed e-CBT as a convenient and time-flexible treatment format that requires attention to factors such as engagement and care-provider interaction (9). Since e-CBT for CWs remains understudied, modifications and other considerations expressed by CWs are critical to continually improving program effectiveness and retention (6).

Meaningful input from participants during clinical trials can enhance care, recruitment, retention, and satisfaction (10). Despite its benefits, this approach is absent in most clinical trials. In the last two decades, only 22 published studies have gathered participant experiences during a trial (11). Consistent measurement of patient experiences during clinical trials is a patient-oriented approach that can improve the implementation of health services (12). Moreover, for digital health tools to contribute meaningfully to improved healthcare delivery, it is important to examine the acceptance of such tools by patients (13). Previous data published by the research team has demonstrated the rich data and missed nuances that are captured when CWs are offered a platform to voice their thoughts and opinions (9). Therefore, the current study aimed to capture experiences with OPTT in a sample of CWs involved in the aforementioned clinical trial. The study aimed to investigate how participants viewed OPTT’s e-CBT modules as well as outline benefits and areas of improvement. The experiences and opinions of CWs will be used to improve OPTT’s therapy content and bridge existing research to practice gaps in this domain (14).

## 2 Materials and methods

### 2.1 Participants and study design

The following mixed-methods study captured the experiences of CWs involved in a clinical trial comparing the efficacy of e-CBT to treatment as usual for individuals diagnosed with major depressive disorder (MDD), generalized anxiety disorder (GAD), or posttraumatic stress disorder (PTSD) (6). Clinical trial participants were current or previous CWs in Canadian federal and provincial facilities in Ontario, Canada, who were between 18 and 55 years of age at the start of the study and diagnosed with MDD, GAD, or PTSD according to the Diagnostic and Statistical Manual of Mental Disorders, 5th Edition (DSM-5) (15). A minimum age of 18 was selected per the province of Ontario’s requirements for CW employment (16). In line with the most common mental health disorders reported amongst CWs (1), three diagnosis-specific e-CBT programs for depression (13 weeks), anxiety (12 weeks), and PTSD (12 weeks) were delivered as weekly sessions and assignments through OPTT. OPTT’s CW-specific programs comprise pre-designed online asynchronous psychotherapy sessions, followed by homework assignments to reinforce the session’s concepts. The sessions can be accessed via computer or mobile phone. The modules integrated examples and case studies obtained from interviews with CWs as well as literature reviews (9, 17). Program participants were instructed to complete their weekly sessions and submit the corresponding homework assignments to their assigned therapist through OPTT. The therapist was responsible for answering questions asynchronously and providing personalized feedback on participants’ session-specific weekly assignments.

The current study used a purposive sample of CWs who had either completed or dropped out of the clinical trial after at least one e-CBT session. A one-year cutoff from the date of participation was established to prevent recall bias (18). Following study completion or dropout, participants were emailed a fillable PDF copy of the survey and asked if they were interested in participating in a one-on-one interview to capture their experiences with the therapy. Those who agreed to take part in the interviews received a 25 CAD Amazon gift card for their time. Participants emailed the completed surveys to the research coordinator after their completion. Surveys and interviews were conducted from March 15, 2022, to August 4, 2023. Participants who were not interested were instructed to notify the research coordinator and their names were removed from the contact list. Ethical clearance was given by the Health Sciences and Affiliated Teaching Hospitals Research Ethics Board at Queen’s University in Kingston, Canada (File: 6029966).

### 2.2 Survey development

The CW post-participation self-reporting survey was adapted from previous surveys developed by the research team through extensive literature reviews, consulting previously validated instruments, and reviewing qualitative data collected at the first stage of the trial (8, 9, 19). Following revisions from members of the research team, the final survey (**Supplementary Data 1**. Survey Results) captured demographic variables (19 items), general impressions (4 items), interactions with technology (3 items), interaction with the care provider (10 items), opinions of the online psychotherapy sessions (9 items), opinions of the exercises and homework (3 items), long term effects of the treatment program (5 items), and module-specific questions (3 items). The remaining six questions were open-ended and enabled participants to provide additional feedback. Due to time constraints and limited scope, the validity and reliability of the final survey were not assessed.

### 2.3 Individual interviews

For an in-depth and nuanced understanding of the insights captured in the survey data, semi-structured interviews were conducted by two trained research assistants (RAs) not involved in the clinical trial. The interviews were no more than 60 minutes in length, and a guide with 28 open-ended questions was used. Before the interviews, the guide was pilot-tested and focused on the participant’s treatment expectations as well as interactions with OPTT, their care provider, and the e-CBT program. All interviews were held over Microsoft Teams, a secure video-conferencing platform, and screen-recorded. Participants were given the option to keep their webcams on or off based on their level of comfort.

### 2.4 Data analysis

The screen-recorded sessions were transcribed *verbatim* by an RA not involved in the interviews. The resulting transcripts were proofread by the RAs conducting the interviews as well as members of the research team involved in analyzing the qualitative data for finalization. All identifying information was omitted from the transcripts and instead followed a labelling convention of gender (W=woman; M=man), group (A=anxiety, D=depression, P=PTSD), completion status (D=dropout; C=completer), and a random number (e.g., MDC1, WPD2). All presented quotes were edited for correct grammar and spelling while preserving meaning and tone.

Surveys were analyzed using Qualtrics (Qualtrics, Provo, UT) statistical analysis software. Missing data were excluded, and each survey item analysis was assessed individually relative to the total responses of the specific item. Response frequency and percentages were used to report categorical variables. Medians, means, ranges, and standard deviations were used for continuous variables. Correlations between demographic characteristics (i.e., age, race, gender, education, marital status, employment status, etc.), completion status, and perceptions of the program were evaluated using Chi-squared tests. Independent samples t-tests and Mann-Whitney U tests were used to assess continuous parametric and non-parametric variables at a significance level of α=0.05. Quantitative analysis was conducted using IBM SPSS Statistics for Mac, version 24 (IBM Corp., Armonk, NY, USA).

This study followed the consolidated criteria for reporting qualitative research (COREQ) checklist to report qualitative findings (20). Textual data from open-ended survey responses and individual interviews were analyzed using thematic analysis methods (21) using NVivo 12 (QSR International Pty Ltd, 2020) by two RAs. Codes generated from the data were organized into themes that described the participants’ opinions and feedback. The final themes were discussed amongst the research team and revised where necessary.

## 3 Results

### 3.1 Participants

The survey was sent to 35 eligible participants. In total, 14 responses were recorded (response rate=40.0%, completion rate=92.86%; n=13/14). On average, the respondents were 37.50 (SD=8.10) years of age, ranging between 25 and 54 years. At the time of the survey, 92.86% of the respondents (n=13/14) were currently employed as correctional workers with an average of 9.90 (SD=7.00) years of experience in their respective roles. Most respondents (64.29 n=9/14) had children (average number of children=2). Among survey respondents, n=9 agreed to take part in the semi-structured interviews. A demographic description of the survey and interview participants can be found in **Table 1**.

**Table 1:** Demographic details of survey respondents (n=14) and allocated e-CBT program.

Category	Survey n (%)	Interviews n (%)
Gender Identity		
*Woman*	7 (50.00)	5 (55.56)
*Man*	7 (50.00)	4 (44.44)
Biological Sex		
*Female*	7 (50.00)	5 (55.56)
*Male*	7 (50.00)	4 (44.44)
Ethnicity		
*White/Caucasian*	13 (92.86)	8 (88.89)
*Other*	1 (7.14)	1 (11.11)
Current Income		
*$50,000 - $74,999*	3 (21.43)	2 (22.22)
*$75,000 - $99,999*	7 (50.00)	3 (33.33)
*Over $100,000*	4 (28.57)	4 (44.44)
Marital Status		
*Married*	11 (78.57)	7 (77.78)
*Single, never married*	2 (14.29)	2 (22.22)
*Common-law*	1 (7.14)	0 (0.00)
Highest Level of Education Completed		
*Bachelor’s degree*	10 (71.43)	5 (55.56)-
*Graduate degree*	2 (14.29)	2 (22.22)-
*Diploma*	1 (7.14)	2 (22.22)-
*High School Diploma*	1 (7.14)	0 (0.00)-
Allocated e-CBT program		
*Anxiety*	5 (35.71)	2 (22.22)
*Depression*	4 (28.56)	3 (33.33)
*PTSD*	5 (35.71)	4 (44.44)

Survey non-respondents (n=21) were, on average, 41.00 (SD=9.179) years old and 81.82% of the sample identified as women (**Supplementary Table 1**. Demographic Information of Survey Non-Respondents). Most non-responders were employed (92.86%), had children (64.86%), and were currently on medication for their mental health (57.14%). Approximately one-quarter (22.73%) of non-respondents completed the e-CBT program in full, significantly less than the survey respondents (p=0.014).

### 3.2 Respondent mental health profile

Most respondents (64.29%; n=9/14) were on medication for their mental health at the time of the survey. When asked to rate the severity of their mental health disorder symptoms, half (50.00%; n=7/14) said their symptoms were somewhat severe, with approximately half (42.15%; n=6/14) saying their symptoms were not severe. One (7.14%; n=1/14) respondent said their symptoms were very severe. Nearly half (46.15%, n=6/13) of the respondents had not previously received any psychotherapy. Those who received therapy either engaged in-person (38.46%; n=5/13) and/or online/telephone delivery of psychotherapy (38.46%; n=5/13). Over half of the respondents (53.85%, n=7/13) had previously received CBT, either in-person (23.08%, n=3/13) or remotely (30.77%, n=4/13).

### 3.3 Treatment preferences

Nearly three-quarters (69.23%; n=9/13) of respondents completed all sessions of OPTT’s e-CBT program. After taking part in the e-CBT program, most respondents (61.54%; n=8/13) preferred to interact with their care provider in person, followed by online video (30.77%; n=4/13) and online-text-only (7.69%; n=1/13). Nearly one-quarter (23.08% n=3/13) had no preference, and one participant (7.69%) was unsure about their preferred method of interacting with their care provider. None of the participants preferred phone calls. Participants were relatively evenly split between the devices they used most to access their e-CBT program on OPTT (computer=57.14%; n=8/14, cellphone=42.86%; n=6/14).

### 3.4 Post-treatment perception

Most respondents (64.29%; n=9/14) felt the program increased their confidence in their ability to manage their mental health symptoms, and all (100.00% n=14/14) felt the e-CBT program was worth their time. An overall positive perception was observed regarding the OPTT platform and satisfaction levels, particularly with the technology and homework assignments. Encouragingly, a large majority (84.62%; n=11/13) of respondents also said they were “likely” to “very likely” to seek treatment again in the future.

Respondents had a positive response towards their care providers, with all respondents ranking their care providers’ degree of empathy displayed to them as either “good” or “very good” (**Table 2**). Moreover, there were positive perceptions of the individual sessions for the online psychotherapy, including the appropriateness of the number of sessions, the amount of time spent on each session, and the order of the sessions (**Table 2**). For diagnosis-specific questions (i.e., depression, anxiety, PTSD), participants generally expressed neutral-positive feelings toward the content and skills covered in the sessions (**Table 3**).

**Table 2:** General impressions of the care provider interactions and the online psychotherapy sessions.

Care Provider Statements	Very Poor	Poor	Neutral	Good	Very Good
*The care provider’s ability to guide through the program*	0	1	1	6	5
*Quality and relevance of the feedback given by the care provider*	0	1	1	4	7
*Ease of understanding the care provider’s feedback*	0	1	0	5	7
*Speed at which the care provider provided feedback*	0	0	0	4	9
*Helpfulness of the care provider*	0	1	1	5	6
*Degree of empathy displayed by the care provider*	0	0	1	2	10
*Degree of comfort in self-disclosing to the care provider*	0	1	0	4	8
*Confidence in the care provider’s knowledge*	0	0	0	4	9
*Availability of the care provider*	0	0	2	2	8
*Overall experience working with the care provider*	0	1	0	6	6
Online Psychotherapy Statements	Very Poor	Poor	Neutral	Good	Very Good
*Clarity of the information presented in the sessions*	0	1	0	5	6
*Helpfulness of the information received in dealing with problems effectively*	0	0	0	7	5
*Relevance of the sessions to experiences with mental health challenges*	1	0	3	4	5
*Appropriateness of the number of sessions*	1	0	2	5	4
*Appropriateness of the order of sessions*	0	0	0	6	6
*Impressions about receiving new sessions once per week*	1	1	3	2	6
*Appropriateness of the time spent on the sessions*	0	1	1	5	5
*Motivation to complete the sessions*	1	1	4	3	4
*Overall impressions of the sessions*	0	1	0	5	6

**Table 3:** Disorder-specific psychotherapy program perceptions (PTSD, Anxiety, & Depression).

Statement	Strongly Disagree	Disagree	Neutral	Agree	Strongly Agree
*The program provided a good overview of my diagnosis*	1	0	5	6	8
*The skills learned helped me manage my symptoms related to my diagnosis*	0	2	6	8	4
*The skills I learned helped me effectively cope with stressful situations*	0	2	6	8	4

### 3.5 ​​Qualitative assessment of the therapy experience

Interviews exploring participants’ experiences of the therapy program revealed five themes: 1) The research study as a support for CWs, 2) User-friendliness of the Online Psychotherapy Tool (OPTT), 3) Tailored therapy experience, 4) Online therapy as an accessible way to normalize experiences and learn coping skills, and 5) Limitations in therapist-client interactions and online format. The themes and associated subthemes are described below.

#### 3.5.1 Theme: the research study as a support for CWs

Participants expressed that the way that this research study was designed and advertised supported their decision to participate and their willingness to seek help. The participants also recognized the uniqueness of the study, which contrasted with other mental health programs that did not focus directly on CWs and their unique needs. Participants mentioned several negative factors in their workplace, including demanding work schedules, violence, and other traumatic experiences, which often resulted in serious consequences. Some participants also found that their managers largely discounted staff attempts to seek out mental health services. For this reason, CWs were glad that management was not involved in advertising this research study. They also explained that mental health symptoms or psychological distress are highly stigmatized and often considered a sign of weakness in their workplace.

*“You should be doing it [advertising the online psychotherapy program] as much as possible because, I hate to say it, but corrections don’t do a lot for their staff at all. I’ve seen too many friends go through this. I’ve seen too many, way too many co-workers take their lives. As a matter of fact, in 25 years as a correctional officer, I’ve seen more correctional officers commit suicide than inmates.” (MPC1)*

#### 3.5.2 Theme: user-friendliness of the Online Psychotherapy Tool (OPTT)

Most participants were content with the platform, mentioning that it met their expectations. The few participants who requested technical support were pleased with the help received. Many participants believed that the platform supported their therapy experience, focusing on its organization and user-friendliness.

*“The graphics were good. The language is easy to understand. There was nothing super difficult, or nothing was super convoluted. And if I needed help, there was a little help chat, which was helpful.” (MAC1)*

#### 3.5.3 Theme: tailored therapy experience

In general, participants had favorable views on the program design. The content flow and organization, the pace of the sessions and assignments, and the details of the different coping techniques positively contributed to their therapy experience. They found their care provider interactions to be helpful and supported their skill-building. Participants also noted the relevance of the examples and case studies used in the sessions, which helped provide more clarity on the types and effects of their traumatic experiences and symptoms.

*“I think it encompassed a good portion of the job. And I feel like for what they were intended to help with, they were good examples to use.” (WAC1)*

#### 3.5.4 Theme: online therapy as an accessible way to normalize experiences and learn coping skills

Participants described several ways the program and its features impacted their lives. Due to demanding work schedules and mental health stigma, taking time off work or finding accessible services was a significant barrier to obtaining mental healthcare. The program’s online format improved access to mental health services and enabled participants to engage in it from the privacy and comfort of their homes, or while taking a break from work. Participants also believed that the program improved their symptom management by teaching helpful coping skills and normalizing their experiences as CWs. Many recognized that after participating in this program, they could assess stressful situations from a different perspective, giving themselves enough time to process unhelpful thoughts instead of reacting to them.

*“I practice challenging my thoughts and approaching my reactions differently, I will continue to do that.” (WDC1)*

#### 3.5.5 Theme: limitations in therapist-client interactions and online format

Some participants expressed difficulties interacting with different elements of the platform, including finding their sessions, responding to, and clearing the notifications, and accessing information that was covered in previous sessions. Additionally, some participants were discontent with the online format. They mentioned that the lack of face-to-face care provider interaction made the therapy program feel impersonal and easier to avoid, that the deadlines and homework were overwhelming at times, that they would have preferred to talk things through rather than write them down, and that they didn’t use the chat system because they wanted more immediate answers to their questions. Moreover, the participants who dropped out of the study expressed that the deadlines and lack of therapist interaction were the main factors in their decision to leave the program.

*“I think not having that face-to-face human element to it, it kind of … it made it very sterile, and it is not the same as actually having a conversation and seeing body language and how that person interacts and is feeling.” (MPD1)*

## 4 Discussion

CWs experience some of the highest rates of mental health disorders and suicidal thoughts and behaviors among PSPs (1, 3). Combined with shift-based work schedules and a stressful work environment, it is prudent to offer accessible mental health care. The current study captured the treatment experiences of CWs enrolled in OPTT, an online therapist-led asynchronous psychotherapy platform. The general responses of participants in the clinical trial were positive, and the majority had a good experience with OPTT, supporting online mental health care as a therapeutic modality.

Although most CWs preferred in-person therapy, they believed that e-CBT was worth their time and increased their confidence in managing their mental health symptoms. Indeed, PSP in other e-CBT trials reported reductions in depression, anxiety, and PTSD symptoms (5). The current study provides context into the aspects of the program that contributed to improved symptoms - namely, learning about different coping techniques, clarity and understanding of their traumatic experiences and resulting symptoms, increased confidence in managing mental health symptoms, and a high perceived likelihood that the positive changes from the therapy would be sustained.

Participants also found the therapists to be knowledgeable and empathetic and supported CWs’ skill-building and guidance through the program. Despite the positive perceptions, most participants preferred in-person and online video interactions with their therapists - a contrast to the asynchronous text-based communication in the present program. The qualitative data provided context to these findings as face-to-face interactions were believed to provide more immediate answers to questions and reduce the impersonal aspects of the program. Moreover, a lack of therapist interaction was one of the contributing factors for non-completers to drop out of the study. Results of a recent study indicated that compared to online mental health treatments, those conducted in person contribute to better therapeutic alliance (22). At the same time, it is important to consider that the evolution of online therapeutic relationships and their influence on treatment outcomes is relatively novel (23). Previous data from the research team demonstrated that individuals with higher depression symptomatology had a preference for in-person therapy, whereas those with lower severity opted for its online counterpart (24). Although most participants in the current study reported experiencing somewhat severe mental health symptoms, future studies will benefit from considering the factors that may influence therapeutic preferences amongst CWs.

In addition to therapist interaction, the current study highlighted profiles evident within CWs seeking online therapy. Most respondents were or had previously received either pharmacotherapy or psychotherapy before the current program. In addition, most of the participants indicated that they would likely seek treatment again in the future. In line with the findings of a previous study, PSPs’ hesitance to engage in help-seeking due to stigma and confidentiality may be offset by the provision of easy-to-use electronic resources, occupation-specific content, and ensuring confidentiality - factors that have been acknowledged in the current program (25). Future studies may also benefit from exploring how the frequency of help-seeking within CWs can influence their perceptions of the treatments offered. Given that homework deadlines were a cited reason for participants to drop out, the current findings also highlight the importance of considering CW work schedules to further improve the ease of access to online therapies. In addition, the qualitative findings shed light on the stigmatizing work environment that may increase CWs’ demands for treatments and programs not affiliated with or offered by their employer. Although it is beyond the scope of this paper, these preliminary findings support the importance of considering how CW work environments impact not just overall help-seeking but the kinds of treatments they are more likely to engage with.

### 4.1 Limitations

Despite its strengths, the study also had several limitations that warrant consideration. Compared to survey respondents, most non-respondents did not complete the e-CBT program. As such, the findings may support more favorable or beneficial views of the program. Although selection bias is a common concern in survey and qualitative studies (26, 27), all OPTT participants, irrespective of their completion status, were engaged in the current study. Differences in the profiles of study participants and non-participants may indicate other factors that influence the willingness of an individual to take part in post-intervention assessments. Second, the sample was relatively homogenous, comprising individuals who were white, cis-gender, and highly educated. The literature is scarce in terms of mental health interventions targeting racialized CWs, which may be due to the low racial diversity that exists in this line of work (28, 29). Accordingly, most related studies either fail to report the ethnicity of their sample or report data primarily from cis-gender, white, and well-educated populations (30–33). Although the content of the e-CBT acknowledged the importance of diversity and inclusion, future studies need to identify effective avenues to enhance equitable treatment accessibility and address any systemic limitations that prevent or discourage racialized CWs from seeking care. While the insights collected from the small sample size provide important insights into CWs’ experiences of e-CBT, the findings should be interpreted with caution. At the same time, the qualitative findings were sufficient in fruitfully outlining areas of strength and improvement in e-CBT, which are necessary for guiding the development of this field.

### 4.2 Summary and conclusions

This study used a survey and semi-structured interviews to investigate the experiences of CWs who participated in a diagnosis-specific online psychotherapy program. Overall, there was a positive perception of the psychotherapy program, the technology, the therapist interactions, and the exercises and homework assignments. Despite the positive response to the online program, most respondents still preferred in-person therapy, reaffirming the need for further improvements to therapeutic rapport within digital mental health interventions. End-users felt more prepared to handle their symptoms post-treatment and felt that the program would have long-term benefits. Nevertheless, participants felt their therapists displayed empathy and were able to develop a good rapport with them, an encouraging note as the therapist interaction was through an asynchronous, written format. Qualitative data revealed stigma in the workplace as a large deterrent to seeking treatment. Future research should investigate how the CW work environment can increase treatment-seeking behaviors.

## Data availability statement

The raw data supporting the conclusions of this article will be made available by the authors, without undue reservation.

## Ethics statement

The studies involving humans were approved by Queen’s University Health Sciences and Affiliated Teaching Hospitals Research Ethics Board. The studies were conducted in accordance with the local legislation and institutional requirements. The participants provided their written informed consent to participate in this study.

## Author contributions

EM: Investigation, Methodology, Writing – original draft, Writing – review & editing. GG: Data curation, Formal analysis, Investigation, Writing – original draft, Writing – review & editing. CS: Formal analysis, Investigation, Writing – original draft, Writing – review & editing. TG: Data curation, Writing – original draft, Writing – review & editing. JJ: Data curation, Writing – original draft, Writing – review & editing. CH: Data curation, Writing – original draft, Writing – review & editing. CP: Project administration, Writing – original draft, Writing – review & editing. MO: Writing – original draft, Writing – review & editing. AS: Writing – original draft, Writing – review & editing. NA: Supervision, Writing – original draft, Writing – review & editing.
