# Supplementary material for: Experiences using an online therapist-guided psychotherapy platform (OPTT) in correctional workers with depression, anxiety, and PTSD
Source: Front Psychiatry. 2024 Apr 23;15:1365746. doi: 10.3389/fpsyt.2024.1365746 (PMC11075154; doi:10.3389/fpsyt.2024.1365746)
Supplement: Supplementary file 1 [file Table_1.docx]

**Supplementary Table 1.** Demographic details of non-respondents (n=21) and their allocated e-CBT program.

| **Category** | **% (n)** |
| --- | --- |
| **Gender Identity (n=11)** | |
| *Woman* | 81.82 (9) |
| *Man* | 18.19 (2) |
| *Other* | 0 (0) |
| *Prefer not to answer* | 0 (0) |
| **Biological Sex (n=16)** | |
| *Female* | 50.00 (8) |
| *Male* | 50.00 (8) |
| *Other* | 0 (0) |
| *Prefer not to answer* | 0 (0) |
| **Ethnicity (n=17)** | |
| *White/Caucasian* | 76.47 (13) |
| *Black* | 0 (0) |
| *Hispanic/Latino* | 0 (0) |
| *Indigenous/Native* | 0 (0) |
| *Middle Eastern* | 0 (0) |
| *Asian* | 0 (0) |
| *Multi-ethnic* | 0 (0) |
| *Unknown* | 0 (0) |
| *Other* | 23.53 (4) |
| **Current Income (n=17)** |  |
| *Under $50,000* | 0 (0) |
| *$50,000 - $74,999* | 5.88 (1) |
| *$75,000 - $99,999* | 64.71 (11) |
| *Over $100,000* | 29.41 (5) |
| **Marital Status (n=17)** | |
| *Married* | 58.82 (10) |
| *Single, never married* | 29.41 (5) |
| *Common-law* | 0 (0) |
| *Divorced* | 0 (0) |
| *Separated* | 0 (0) |
| *Widowed* | 0 (0) |
| *Other* | 11.76 (2) |
| **Highest level of education completed (n=17)** | |
| *Diploma* | 47.06 (8) |
| *Bachelor’s degree* | 41.18 (7) |
| *High School Diploma* | 5.88 (1) |
| *Graduate Degree* | 5.88 (1) |
| *Advanced Diploma* | 0 (0) |
| *Other* | 0 (0) |
| **Allocated e-CBT program (n=21)** |  |
| *Anxiety* | 47.62 (10) |
| *PTSD* | 33.33 (7) |
| *Depression* | 19.05 (4) |
